# Medikamentenversorgung und Polypharmazie in der Langzeitpflege: Eine Übersicht zu möglichen Interventionen und die Frage nach einem funktionierenden Konzept

**DOI:** 10.1007/s00391-024-02340-1

**Published:** 2024-08-13

**Authors:** Fabian Graeb, Bianca Berger, Frank Alf, Petra Reiber, Gundula Essig, Reinhold Wolke

**Affiliations:** Institut für Gesundheits- und Pflegewissenschaften, Hochschule Esslingen, Flandernstraße 101, 73732 Esslingen, Deutschland

**Keywords:** Stationäre Langzeitpflege, Arzneimittelbedingte Probleme, Interprofessionelle Zusammenarbeit, Potenziell inadäquate Medikamente, Medikamentenmanagement, Residential nursing homes, Drug-related problems, Interprofessional collaboration, Potentially inappropriate medication, Medication management

## Abstract

**Hintergrund:**

Polypharmazie und daraus resultierende Probleme führen zu erheblichen Belastungen bei den Betroffenen. Darüber hinaus lassen sich erhebliche Probleme bei der Medikamentenversorgung feststellen.

**Fragestellung:**

Welche Interventionen und Programme zur Optimierung der Medikamentenversorgung liegen für die stationäre Langzeitpflege vor, und mit welchen Umsetzungsproblemen ist zu rechnen?

**Material und Methode:**

Literaturrecherche zu Interventionsstudien, die in stationären Pflegeeinrichtungen in Deutschland durchgeführt wurden, mit dem Fokus auf der Optimierung der Medikamentenversorgung.

**Ergebnisse:**

Sechs Programme mit Evaluationsergebnissen konnten identifiziert werden. Obwohl der Ansatz meist multimodal ist und mehrere Interventionsbereiche umfasst, wie Medikamentenbewertungen, Fort- und Weiterbildung sowie die Entwicklung von Hilfsmitteln, sind die Ergebnisse größtenteils enttäuschend. Lediglich in einer Studie konnten signifikante Auswirkungen auf die Gesamtzahl der Verschreibungen, bestimmte Medikamentengruppen und Outcome-Parameter wie Krankenhauseinweisungen belegt werden, wobei hierfür ein Selektionsbias zumindest mitverantwortlich sein könnte. Die größten Schwierigkeiten bestehen in der Umsetzung interdisziplinärer Zusammenarbeit und der Anwendung der in Reviews formulierten Medikamentenempfehlungen durch die zuständigen Ärzt*innen. Gleichzeitig wird die zentrale Rolle der Pflegenden im Gesamtprozess zu wenig beachtet und aktiv gefördert, was ein weiterer Grund für die Schwierigkeiten bei der Umsetzung in der Praxis sein könnte.

**Schlussfolgerungen:**

Es zeigen sich fast keine signifikanten Veränderungen als Folge der in den gesichteten Studien durchgeführten Interventionen. Vor allem die interprofessionelle Kooperation, speziell die Kompetenzen der Pflegenden und die Zurückhaltung aufseiten der Ärzt*innen, müssten hierbei vermutlich noch stärker in den Blick genommen werden.

**Zusatzmaterial online:**

Zusätzliche Informationen sind in der Online-Version dieses Artikels (10.1007/s00391-024-02340-1) enthalten.

## Abstract (en)

**Background:**

Polypharmacy and the resulting problems lead to considerable consequences for those affected. There are also considerable problems with the medication management.

**Objective:**

Which interventions and programs for optimizing the supply of medication are available for nursing homes and which implementation problems can be expected?

**Material and method:**

A literature search was carried out for interventional studies in nursing homes in Germany, with a focus on improving medication safety.

**Results:**

A total of six programs were identified for which evaluation results are available. Despite a mostly multimodal approach with several pillars of intervention (e.g., medication reviews, further education and training, development of aids), the results are largely disappointing. The effects on the number of prescriptions in general, specific medication groups or outcome parameters such as hospital admissions could only be shown in one study, whereby, selection bias could also be at least partly responsible for this. Interdisciplinary collaboration and the implementation of medication recommendations formulated in reviews by the responsible physicians are the main problem areas. At the same time, too little attention is paid to the central role of nurses in the entire process and they are not actively promoted enough. This could be one of the reasons for the difficulties in implementation in practice.

**Conclusion:**

There are nearly no significant changes as a result of the interventions implemented in the studies reviewed. In particular, interprofessional cooperation, especially the skills of nurses and the reluctance on the part of physicians, should probably be given more attention.

## Hintergrund

Polypharmazie, die Einnahme vieler Medikamente, ist ein Phänomen, das besonders bei älteren Bewohner*innen der stationären Langzeitpflege verbreitet ist. Dieses Phänomen ist nicht einheitlich definiert, überwiegend ist aber die Einnahme von 5 oder mehr verschiedenen Medikamenten am Tag gemeint [18]. Polypharmazie geht dabei oft mit erheblichen negativen Folgeerscheinungen einher und stellt die medizinische Versorgung vor große Herausforderungen.

In einer Population von 918 psychisch erkrankter Pflegeheimbewohner*innen werden z. B. im Schnitt 7,1 (SD ± 3,2) verschiedene Medikamente täglich eingenommen [21], in einer weiteren Untersuchung von 495 Bewohner*innen sind es gar 10,8 (SD ± 4,7) Medikamente [7]. Die vermehrte Medikamenteneinnahme im Alter ist häufig auf eine zunehmende Multimorbidität zurückzuführen, was nicht nur auf Bewohner*innen der Langzeitpflege zutrifft. So werden 77,5 % der Personen in der Altersgruppe 80+ als multimorbid eingestuft [19]. Tatsächlich sind Personen mit einer Multimorbidität auch häufiger von fehlerhaften Verschreibungen und den Folgen einer Polypharmazie betroffen [12].

Eine Polypharmazie und die Verschreibung bestimmter Medikamentengruppen wie u. a. zentralnervös dämpfende Arzneimittel werden mit Stürzen [1, 15] und einer größeren Gefahr von Medikamenteninteraktionen in Verbindung gebracht [14]. Polypharmazie ist außerdem mit arzneimittelbedingten Hospitalisierungen assoziiert, wobei hier u. a. Antikoagulanzien, β‑Blocker und ACE-Hemmer auffällig gehäuft zu finden sind [20, 26]. Im Zusammenhang mit u. a. Antidepressiva, Glukokortikoiden und Antiepileptika wird von ungewollter Gewichtszunahme berichtet, während Wirkstoffe wie z. B. Zonisamid und Topiramat mit einer Gewichtsabnahme assoziiert sind [17]. Auch die Einnahme von im Alter potenziell ungeeigneten Medikamenten („potentially inappropriate medication“, PIM) geht mit einem erhöhten Sturzrisiko [2], häufigeren Auftreten von Frailty [9] und erhöhtem Risiko für schwerwiegenden Medikamenteninteraktionen einher [21].

### Herausforderungen im Setting stationäre Langzeitpflege

Die eingangs dargestellten Studien zeigen eine grundlegend komplexe Verordnungssituation, mit häufiger (ausgeprägter) Polypharmazie, bei gleichzeitiger Multimorbidität und ausgeprägter Pflegebedürftigkeit bei den Bewohner*innen. Dies führt an sich schon, wie dargelegt, zu erhöhten Risiken. Die Besonderheiten des Settings stationäre Langzeitpflege tragen dann aber zusätzlich zu Schwierigkeiten beim Medikamentenmanagement bei. So ist für die ärztliche Versorgung der Bewohner*innen eine Vielzahl an Hausärzt*innen zuständig, die wiederum in unterschiedlichem Maße Hausbesuche durchführen. Dies erhöht die Anzahl an Schnittstellen und damit auch die Komplexität der Kommunikation zwischen den Beteiligten [10]. Die Apotheken wiederum sind häufig lediglich Lieferanten der Medikamente und in den Prozess der Medikamentenversorgung nicht weiter eingebunden [6]. Pflegende sind an der Initiierung zur Medikamentenverordnung oftmals beteiligt, stellen teilweise die Medikamente, verabreichen diese und sollen Therapieerfolg wie auch unerwünschte Wirkungen (UAW) beobachten. Allerdings werden die Pflegenden über Indikationen, erhoffte Therapiewirkungen und mögliche UAW nicht ausreichend informiert, was eine gezielte Krankenbeobachtung erheblich erschwert [8]. Es zeigen sich im Gesamten massive strukturelle Defizite bei der Kommunikation und Kooperation zwischen den am Medikationsprozess Berufsgruppen, auch an den Schnittstellen stationär-ambulant [6], wodurch es häufiger zu arzneimittelbedingten Problemen kommt [11]. Auch das pharmakologische Fachwissen wird als verbesserungswürdig eingeschätzt [8].

## Fragestellung und Methodik

Die Frage ist daher, welche Konzepte und Interventionen zur Verbesserung der Situation der Betroffenen beitragen könnten, und welche Schwierigkeiten bei der praktischen Umsetzung zu erwarten sind. Hierfür wurde eine Literaturrecherche, die sich auf Interventionsstudien in stationären Pflegeeinrichtungen konzentrierte, (Hinweis: kein systematisches Review), durchgeführt. Gesucht wurde in den Datenbanken PubMed und CINAHL. Als Suchbegriffe dienten „polypharmacy“ in Kombination mit „nursing home“, „long term care“ und „residential care“ (Zusatzmaterial online). In die Auswertung einbezogen wurden dann lediglich Interventionsstudien in Pflegeeinrichtungen in Deutschland (ab 2003), da es hier spezifische Bedingungen gibt (wie gesetzliche Grundlagen von SGB V und XI, Qualifikationsmix in der pflegerischen Versorgung etc.), die eine Übertragung von Erkenntnissen aus anderen Ländern erschweren.

Ausgehend von den detektierten Projekten wurde nach zugehöriger grauer Literatur gesucht (Google Scholar, Google), um Schwierigkeiten bei der Umsetzung und mögliche Stolpersteine identifizieren zu können, die ggf. in der Primärliteratur nicht ausreichend beschrieben wurden. Auch wenn etwa keine veröffentlichten Ergebnisse von publizierten Studienskizzen gefunden werden konnten, wurde unter Zuhilfenahme des Projekttitels eine Google-Suche durchgeführt, um beispielsweise Projektberichte zu identifizieren.

## Ergebnisse

In der stationären Langzeitpflege in Deutschland konnten nur 6 Studien, die konkrete Ergebnisse lieferten, gefunden werden. Die Auswahlkriterien der Recherche und detaillierte Ergebnisse können im Zusatzmaterial online eingesehen werden.

Im interdisziplinär aufgebauten Projekt *Arzneimitteltherapie in Alten- und Pflegeheimen* von [23] wurde auf Basis einer ersten prospektiven Querschnitterhebung (11 Einrichtungen, 778 Bewohner*innen) eine Intervention entwickelt, die auf ihre Umsetzbarkeit geprüft werden sollte. Die Intervention basierte auf strukturierten Fortbildungsangeboten für die zuständigen Allgemeinmediziner*innen, Schulungen des Pflegepersonals zur Sensibilisierung und frühzeitigem Erkennen von unerwünschten Arzneimittelwirkungen (UAW) sowie arzneimittelbedingten Problemen (AbP) und Schulungen zur Erkennung von AbP und den damit zusammenhängenden Interventionsmöglichkeiten für die heimversorgenden Apotheker*innen. Jede Einrichtung sollte zudem ein AMTS-Team (AMTS: Arzneimitteltherapiesicherheit) mit einer Pflegekraft und einer/m Apotheker*in bilden, die mithilfe von Fallbeispielen geschult wurden. Auch Hilfsmittel für die tägliche Umsetzung, wie die AMTS-Karte für einen schnellen inhaltlichen Überblick, wurden erstellt [23]. Die entwickelten Interventionen wurde dann in 4 Einrichtungen mit 42 Proband*innen getestet und als „machbar“ eingeschätzt. Es gab jedoch Schwierigkeiten bei der Einbindung der Hausärzte*innen.

Aufbauend auf diesen Erfahrungen wurde die Intervention optimiert und die Studie *Arzneimitteltherapiesicherheit bei Patienten in Einrichtungen der Langzeitpflege (AMTS-AMPEL)* durchgeführt. Primäres Outcome-Kriterium war die Reduktion von UAW. Anhand eines Vorher-nachher-Designs konnte ein signifikanter Rückgang vermeidbarer UAW nach 6 und 12 Monaten Intervention aufgezeigt werden. Gleichzeitig stieg aber die Häufigkeit von nichtvermeidbaren UAW etwas an. Die Intervention konnte nicht immer vollständig umgesetzt werden. Obwohl das Programm von den Mitarbeitenden der teilnehmenden Einrichtungen als insgesamt positive Erfahrung gewertet wurde, fehlte nach Projektende oft die Bereitschaft, mit den bereitgestellten Materialien und Verfahren nach Projektabschluss weiterzuarbeiten [24].

Die Cluster-randomisierte HIOPP-3-iTBX-Studie baut wiederum auf den beiden zuvor genannten Projekten auf. Die getestete Intervention fußt auf 4 Elementen: Medikationsreviews durch spezifisch geschulte Apotheker*innen, Fortbildungen für Pflegekräfte und Hausärzt*innen, eine Toolbox zur AMTS (Entlass- sowie Visitentool, AMTS-Karte etc.) und Change-Management-Seminare für Pflegende, Ärzt*innen und Apotheker*innen. Untersucht wurde der primäre Endpunkt Verordnung von PIM und/oder mindestens 2 Neuroleptika. Als sekundäre Endpunkte wurden Sturzinzidenz, Hospitalisierungen, Lebensqualität und Gesundheitskosten evaluiert. Es zeigten sich bei Primär- und Sekundärendpunkten keine signifikanten Effekte in der Interventionsgruppe [13].

Die Forschungsgruppe um Wolf-Ostermann hat im Projekt *MADRIC* ein IT-gestütztes Monitoring-System zur Vermeidung von UAW entwickelt und getestet. Die Einrichtungen des Berliners Projekts wurden rekrutiert, da sie speziell angestellte Ärzte*innen haben, die die Bewohner*innen betreuen. Eine Einrichtung diente als Interventions-, eine als Kontrolleinrichtung. Zielvariablen waren die gesundheitsbezogene Lebensqualität (Instrument Short Form 36), der Pflegebedarf (Resident Assessment Instrument 2.0, RAI), Funktionsfähigkeit (Aktivitäten des täglichen Lebens, ADL), kognitive Fähigkeiten (entnommen aus dem RAI) und Sturzrisiko (Stratify Score). Nebenparameter umfassten die Komorbidität (Elixhauser Comorbidity Index), die Ernährungssituation (Nutritional Risk Screening) und aus dem RAI abgeleitete Faktoren, wie Dekubitus, Schmerzen, Depression, Anhedonie und Aggressivität. In die Evaluation gingen 48 Bewohner*innen der Interventions- und 72 der Kontrolleinrichtung ein. Die vom digitalen System den Ärzt*innen angezeigten Meldungen zu möglichen Problemen wurden allerdings in 95,6 % der Fälle abgelehnt; eine Anpassung der Therapie wurde aus verschiedenen Gründen nicht vorgenommen, selbst wenn es sich lediglich um Vorschläge zur Dosisanpassungen handelte. Bei der Erhebung der Outcome-Parameter zeigten sich keine signifikanten Unterschiede [25].

In der Interventionsstudie *InTherAKT* sollte die interprofessionelle Zusammenarbeit verbessert werden, um so eine angemessene Medikation und ein verbessertes gesundheitsbezogenes Outcome bei den Bewohner*innen zu erreichen. Hierfür wurden umfangreiche Bildungsmaßnahmen für die beteiligten Berufsgruppen und interprofessionelle Medikation-Reviews in einer stationären Einrichtung etabliert. Im Ergebnis führte die Intervention zu einer besseren Medikamentenversorgung, weniger schwerwiegenden Arzneimittelinteraktionen und seltener auftretendem agitiertem Verhalten bei den Bewohner*innen. Es wurden keine Effekte auf die Entwicklung der Kognition, eines Delirs, der Mobilität und die Gesamtzahl der verordneten Medikamente beobachtet [16].

Das Projekt *Optimierte Arzneimittelversorgung für pflegebedürftige geriatrische Patienten (OAV)* [5] zielte mit einer neuen Form der Kooperation zwischen Pflegekräften, Ärzte*innen und Apotheker*innen sowie eines klinisch geprüften EDV-gestützten Risikomanagements darauf ab, die Versorgung von ambulanten oder stationären Pflegebedürftigen oder Patienten*innen, zu verbessern. Teile der betrieblichen und Hochschulausbildung in der praktischen Geriatrie wurden von den 3 Berufsgruppen gemeinsam absolviert. Das Studiendesign wird als eine multizentrische, nichtrandomisierte, 2‑armige Kohortenstudie beschrieben. Teilnehmen konnten Personen, die bei den Krankenkassen AOK Nordost, IKK Brandenburg und Berlin bzw. VIACTIV versichert waren. Die Auswahl der Interventionsgruppe erfolgte prospektiv, die der Kontrollgruppe retrospektiv. Insgesamt konnten 1325 Personen (110 ambulant, 1215 stationär versorgt) in die Interventionsgruppe eingeschlossen werden. Primärer Endpunkt der Evaluation waren UAW, sekundäre Endpunkte Krankenhausweinweisungen, Anzahl verordneter PIM und das Vorliegen einer Polypharmazie. Die Inzidenz von UAW sank signifikant um 27,5 %, die Krankenhauseinweisungen um 17,5 %, während sich das Ausmaß der Polypharmazie und die Anzahl verordneter PIM nur bei den Pflegeheimbewohner*innen signifikant reduzieren ließ [5].

## Stolpersteine und Lösungsansätze

Die Studien- und Evidenzlage ist als heterogen zu bezeichnen. Auf der einen Seite kann die HIOPP-3-iTBX-Studie mit einem Cluster-randomisierten Design als die wissenschaftlich hochwertigste Arbeit betrachtet werden, die zudem auf den Erfahrungen aus 2 Vorgängerprojekten und daraus erarbeiteten komplexen Interventionspaketen basiert. Gleichzeitig zeigen sich gerade hier keine signifikanten Verbesserungen. Die Autor*innen der Studie weisen zudem auf konkrete Probleme hin. So wurden etwa nur selten Empfehlungen der Apotheker*innen in den Reviews durch die Hausärzt*innen übernommen. Von 939 Medikationen mit Optimierungsbedarf kam es nur bei 153 zu einer Veränderung der Medikation. Dies hat laut den Autor*innen verschiedene Gründe, die auch auf eine komplexe Gemengelage aus Multimorbidität und Erwartungen vonseiten der Bewohner*innen, Angehörigen und Pflegenden zurückzuführen sind. Gleichzeitig wird auf die Wichtigkeit persönlicher Kontakte und Zusammenarbeit verwiesen, die nicht gegeben war [13]. Auch die Ergebnisse der MADRIC-Studie deuten an, dass es für die behandelnden Ärzt*innen offenbar eine erhebliche Hürde darstellt, die verordnete Medikation zu ändern [25]. Woran dies im Detail jeweils liegen könnte, wird in den Arbeiten als nicht endgültig beurteilbar beschrieben [23]. Als mögliche Gründe könnten Zeitmangel speziell der Hausärzt*innen oder auch Unwillen, komplexe Medikationen zu ändern, v. a. wenn die Anordnungen durch Fachärzt*innen anderer Disziplinen stammen, infrage kommen. Schon in der Studie AMTS-AMPEL hatte sich aber gezeigt, dass die Treffen des AMTS-Teams relativ selten stattfanden und das Interesse an der Intervention bei Pflegenden und Apotheker*innen höher war als bei den beteiligten Ärzt*innen [24]. Auch im Bericht zum OAV-Projekt wird von Schwierigkeiten bei der Kooperation zwischen den Pflegeeinrichtungen und Ärzt*innen berichtet, die auf die vorherrschenden Versorgungsstrukturen zurückgeführt werden, ohne diese aber konkreter zu benennen [5]. In einer Gruppendiskussion mit den beteiligten Hausärzt*innen im Projekt AMTS-AMPEL wird aber konkret auf Probleme bei der Dokumentation in den Einrichtungen (Papier vs. digital, kein direkter Zugriff durch Ärzt*innen), Konflikte mit den Pflegenden bei unterschiedlichen Standpunkten, teilweise schwierige Zusammenarbeit mit den Apotheken sowie dem fehlenden Austausch mit Fachärzt*innen bei Medikationsänderungen verwiesen [24].

Die Studie OAV zeigt (als Einzige) positive Ergebnisse. Hier zeigten sich in Bezug auf die Bewohner*innen stationärer Pflegeeinrichtungen signifikante Verbesserungen der Arzneimitteltherapie über alle Outcome-Parameter hinweg. Allerdings bestehen erhebliche methodische Einschränkungen durch die fehlende Randomisierung, was die Ergebnisse möglicherweise maßgeblich verzerrt haben könnte. Diejenigen, die sich für die Intervention gemeldet haben, sind ggf. schon zuvor für mögliche Probleme hinsichtlich der Medikation ihrer Angehörigen sensibilisiert gewesen. Dementsprechend würden Bedenken und Beschwerden möglicherweise auch eher und mit Nachdruck berichtet werden, was wiederum erst eine Reaktion auf ärztlicher Seite in Form einer Medikationsveränderung wahrscheinlich macht.

### Interprofessionelle Kooperation

Interessant erscheint beim OAV-Programm, dass in diesem die interprofessionelle Verzahnung stark fokussiert wurde, sodass auch Teile der Ausbildung gemeinsam gestaltet wurden [5]. Auch die InTherAKT-Studie fokussierte auf eine Verbesserung der Zusammenarbeit. Wenngleich diese Studie keine Kontrollgruppe beinhaltete, konnten dennoch Verbesserungen bei der individuellen Medikamentenversorgung für einen Teil der Kohorte festgestellt werden (bei hohem Score im Medication Appropriateness Index, MAI). Die Bedeutung einer gelingenden Zusammenarbeit wird in allen anderen Studiengruppen erkannt, verbunden mit erheblichen Anstrengungen diese beispielsweise in interprofessionellen Medikamenten-Reviews zusammenzuführen. Gleichzeitig wird aber genau diese Kooperation auch als größter Stolpersteine in den jeweiligen Diskussionen beschrieben. Woran das im Einzelnen liegt, ist schwer zu ermitteln. Zeit- und Personalmangel spielen sicherlich eine Rolle, wie auch die räumliche Trennung der einzelnen Akteur*innen. Ein regelmäßiges Forum zu etablieren, möglichst niederschwellig, etwa per Online-Meeting, könnte dazu beitragen, eine Optimierung zu ermöglichen. Ob eine ausreichende personelle Ausstattung der in den Studien beteiligten Einrichtungen gegeben war und ggf. deren Einfluss auf die Ergebnisse (*beachte*: in Fall- und Kontrollgruppe), lässt sich allerdings nicht abschätzen.

Ein Aspekt, der möglicherweise bislang zu wenig beachtet wird, ist die Rolle der Pflegenden. Es wird notwendig sein, die pflegerische Kompetenz stärker einzubinden. Zum einen stammen notwendige Therapieinformationen in der stationären Langzeitpflege hauptsächlich von Pflegenden [10]. Zum anderen verbessert eine Fachweiterbildung in Pain Nursing und Geriatrie nachweislich die positiven Effekte auf die Therapiesteuerung [22]. Dies bedeutet im Umkehrschluss, es werden fachlich gut (weiter)gebildete Pflegekräfte benötigt, weil nur dann eine Kommunikation auf Augenhöhe mit anderen Akteur*innen möglich ist, die Therapiebeobachtung gelingt und die entsprechenden Informationen so weitergegeben werden, dass auch ein Nutzen für die Betroffenen wahrscheinlich wird. Auch hier kann wieder auf das OAV-Programm, bei dem dieser (gemeinsame) Bildungsaspekt stark fokussiert wurde, verwiesen werden.

### Software-Unterstützungssysteme

Ein noch immer relativ neues Thema stellen Software-Unterstützungssysteme dar, v. a. in Verknüpfung mit künstlicher Intelligenz. Gerade, was das frühzeitige Erkennen von potenziellen Problemen betrifft, bieten diese Instrumente ein enormes Potenzial. So wurde in der AMBER-Studie ein Algorithmus zur Erkennung wahrscheinlicher arzneimittelbedingter Probleme (AbP) für die stationäre Langzeitpflege entwickelt [3]. In einem ersten Anwendungstest mit 73 Pflegeheimbewohner*innen konnten 459 potenzielle AbP identifiziert werden, im Schnitt 6,3/Bewohner*in [4].

Solche Entwicklungen in die Praxis zu implementieren, generiert wiederum eigene Schwierigkeiten. Im Zusammenhang mit der MADRIC-Studie wird z. B. von Software- und Schnittstellenproblemen v. a. mit der Software des bestehenden Dokumentationssystems berichtet, insbesondere nach Software-Updates. Zudem werden zu viele Meldungen bemängelt, die auf das wesentliche beschränkt werden sollten [25].

Aus den Berichten geht aber nicht eindeutig hervor, ob es sich bei den zahlreichen Systemalarmen tatsächlich um unwichtige Meldungen ohne klinische Bedeutung handelt. Möglicherweise ist dieser Hinweis ein Ausdruck der Überforderung, da Verordnungen mit oftmals 10 oder mehr Medikamenten eben mit vielen potenziellen AbP einhergehen und damit mit vielen Alarmen.

Ob solche Software-Lösungen tatsächlich etwas an der Verordnungspraxis ändern, ist unklar. Eine Ärztin in der oben genannten MADRIC-Studie beschreibt zwar, dass ihr Verschreibungsverhalten nun kritischer wäre, was sich aber nicht in den Daten niederschlägt. Plausibel erscheint es dennoch, dass solche technischen Unterstützungsangebote hilfreich sein können. Allerdings sind hierzu randomisierte kontrollierte Studien erforderlich. Gleichzeitig ist davon auszugehen, dass der Nutzen überschaubar bleibt, wenn sich die Kooperation der beteiligten Professionen nicht verbessert.

## Fazit für die Praxis

- Es gibt bislang keine Interventionen oder Programme, die eine signifikante Wirksamkeit gezeigt hätten und damit als Goldstandard oder Best practice herangezogen werden könnten. Es mangelt v. a. an der umfassenden Umsetzung der gesichteten Konzepte, was eine endgültige Einschätzung erschwert.
- Neben ausreichenden Personalressourcen sind pharmakologische Weiterbildung und interprofessionelle Verzahnung vermutlich der Schlüssel für ein verbessertes Medikamentenmanagement.
- Die Pflege muss fachlich und organisational gestärkt werden, damit sie ihrer Schlüsselrolle im Medikationsprozess gerecht werden kann.
- Zukünftige Forschung sollte die Argumente und Barrieren für die ärztliche Umsetzung von Medikamentenempfehlungen stärker in den Blick nehmen.

## Supplementary Information

Dokumentation der Recherche

## References

[1] Behrendt S, Schwinger A, Tsiasioti C, Stammann C, Willms G, Hasseler M, Studinski E, Özdes T, Krebs S, Klauber J (2021) Multisektorale Schnittstelle: Hospitalisierungen von Pflegeheimbewohnenden mit Schwerpunkt Sturz. In: Klauber J, Wasem J, Beivers A, Mostert C (Hrsg) Krankenhaus-Report 2021. Springer, Berlin, Heidelberg, S 249–266

[2] Berdot S, Bertrand M, Dartigues J‑F, Fourrier A, Tavernier B, Ritchie K, Alpérovitch A (2009) Inappropriate medication use and risk of falls—A prospective study in a large community-dwelling elderly cohort. BMC Geriatr 9(1):1 19627577 10.1186/1471-2318-9-30 PMC2721838

[3] Erzkamp S, Rose O (2018) Development and evaluation of an algorithm-based tool for Medication Management in nursing homes: the AMBER study protocol. BMJ Open 8(4):e19398 29678967 10.1136/bmjopen-2017-019398 PMC5914904

[4] Erzkamp S, Köberlein-Neu J, Rose O (2021) An Algorithm for Comprehensive Medication Management in Nursing Homes: Results of the AMBER Project. Drug Saf 44(3):313–325 33128697 10.1007/s40264-020-01016-0

[5] Fahrentholz J, Gorny C, Hanke F‑C, Heppner HJ, Langenberger B, siegel M, Vogt V (2023) Optimierte Arzneimittelversorgung für pflegebedürftige geriatrische Patienten. Ergebnisbericht gemäß Nr. 14.1 ANBest-IF

[6] Glaeske G (2017) Apotheken und Pharmaindustrie. In: Brandhorst A, Hildebrandt H, Luthe E‑W (Hrsg) Kooperation und Integration – das unvollendete Projekt des Gesundheitssystems. Springer, Wiesbaden, S 231–243

[7] Graeb F, Jüttner C, Berger B, Wolke R, Reißner J, Essig G, Reiber P (2023) Einschätzung des Risikos für arzneimittel-bezogene Probleme bei Pflegeheimbewohner*innen. Eine retrospektive Analyse von Routinedaten. Z Gerontol Geriatr 56(8):673–678 36577859 10.1007/s00391-022-02152-1 PMC10709222

[8] Grewe AH, Blättner B (2017) Arzneimitteltherapiesicherheit (AMTS) in der ambulanten und der stationären Langzeitpflege. Optionen und Hindernisse für den Beitrag von Pflegekräften. PflWiss 19(11/12):557–564

[9] Herr M, Sirven N, Grondin H, Pichetti S, Sermet C (2017) Frailty, polypharmacy, and potentially inappropriate medications in old people: findings in a representative sample of the French population. Eur J Clin Pharmacol 73(9):1165–1172 28601963 10.1007/s00228-017-2276-5

[10] Jaehde U, Thürmann P (2018) Arzneimitteltherapiesicherheit bei Heimbewohnern. Bundesgesundheitsblatt Gesundheitsforschung Gesundheitsschutz 61(9):1111–1118 30083948 10.1007/s00103-018-2796-x

[11] Jaehde U, Thürmann PA (2012) Arzneimitteltherapiesicherheit in Alten- und Pflegeheimen. Z Evid Fortbild Qual Gesundhwes 106(10):712–716 23217723 10.1016/j.zefq.2012.10.021

[12] Jungo KT, Ansorg A‑K, Floriani C, Rozsnyai Z, Schwab N, Meier R, Valeri F, Stalder O, Limacher A, Schneider C, Bagattini M, Trelle S, Spruit M, Schwenkglenks M, Rodondi N, Streit S (2023) Optimising prescribing in older adults with multimorbidity and polypharmacy in primary care (OPTICA): cluster randomised clinical trial. BMJ 381:e74054 37225248 10.1136/bmj-2022-074054 PMC10206530

[13] Junius-Walker U, Krause O, Thürmann P, Bernhard S, Fuchs A, Sparenberg L, Wollny A, Stolz R, Haumann H, Freytag A, Kirsch C, Usacheva S, Wilm S, Wiese B (2021) Drug Safety for Nursing-Home Residents—Findings of a Pragmatic, Cluster-Randomized, Controlled Intervention Trial in 44 Nursing Homes. Dtsch Ärztebl Int 118(42):705–712 34366004 10.3238/arztebl.m2021.0297 PMC8767148

[14] Lion S, Evrard P, Foulon V, Spinewine A (2023) Drug-drug interactions in nursing home residents: analysis from the COME-ON trial. Age Ageing 52(1) 10.1093/ageing/afac278 36633299

[15] Lippert T, Maas R, Fromm MF, Luttenberger K, Kolominsky-Rabas P, Pendergrass A, Gräßel E (2020) Einfluss zentralnervös dämpfender Arzneimittel auf Stürze mit Verletzungsfolgen bei Menschen mit Demenz in Pflegeheimen. Gesundheitswesen 82(1):14–22 31962367 10.1055/a-1071-7911

[16] Mahlknecht A, Krisch L, Nestler N, Bauer U, Letz N, Zenz D, Schuler J, Fährmann L, Hempel G, Flamm M, Osterbrink J (2019) Impact of training and structured medication review on medication appropriateness and patient-related outcomes in nursing homes: results from the interventional study InTherAKT. BMC Geriatr 19(1):257 31533630 10.1186/s12877-019-1263-3 PMC6749664

[17] May M, Engeli S (2020) Gewichtsänderung als unerwünschte Arzneimittelwirkung. Adipositas – Ursachen Folgeerkrankungen Ther 14(03):133–139

[18] Pazan F, Wehling M (2021) Polypharmacy in older adults: a narrative review of definitions, epidemiology and consequences. Eur Geriatr Med 12(3):443–452 33694123 10.1007/s41999-021-00479-3 PMC8149355

[19] Puth M‑T, Weckbecker K, Schmid M, Münster E (2017) Prevalence of multimorbidity in Germany: impact of age and educational level in a cross-sectional study on 19,294 adults. BMC Public Health 17(1):826 29047341 10.1186/s12889-017-4833-3 PMC5648462

[20] Schurig AM, Böhme M, Just KS, Scholl C, Dormann H, Plank-Kiegele B, Seufferlein T, Gräff I, Schwab M, Stingl JC (2018) Adverse Drug Reactions (ADR) and Emergencies. Dtsch Ärztebl Int 115(15):251–258 29735005 10.3238/arztebl.2018.0251 PMC5949373

[21] Specka M, Groll M, Scherbaum N, Wiltfang J, Benninghoff J (2022) Risikoidentifikation bei Polypharmazie in einer Pflegeheimpopulation. Z Gerontol Geriatr 55(3):231–238 33570659 10.1007/s00391-021-01850-6 PMC9064868

[22] Stolz R, Krause O, Junius-Walker U, Thürmann P, Fuchs A, Wilm S, Wollny A, Rebentisch F, Wiese B, Joos S, Haumann H (2023) The role of qualification and quality management in the prescription of antipsychotics and potentially inappropriate medication (PIM) in nursing home residents in Germany: results of the HIOPP-3-iTBX study. Aging Clin Exp Res 35(10):2227–2235 37550560 10.1007/s40520-023-02513-9 PMC10520111

[23] Thürmann P, Jaehde U (2011) Arzneimitteltherapiesicherheit in Alten- und Pflegeheimen: Querschnittsanalyse und Machbarkeit eines multidisziplinären Ansatzes. Abschlussbericht Bundesministerium für Gesundheit. https://www.bundesgesundheitsministerium.de/fileadmin/Dateien/5_Publikationen/Gesundheit/Berichte/Abschlussbericht_Arzneimitteltherapiesicherheit_in_Alten-_und_Pflegeheimen_Querschnittsanalyse_und_Machbarkeit_eines_multidisziplinaeren_Ansatzes.pdf. Zugegriffen: 7. Aug. 2020

[24] Thürmann P, Jaehde U (2016) ArzneiMitteltherapiesicherheit bei Patienten in Einrichtungen der Langzeitpflege (AMTS-AMPEL). Eine prospektive Interventionsstudie. Bonn, Wuppertal

[25] Wolf-Ostermann K, Schmidt A, Gräske J (2016) IT-gestütztes Monitoring von unerwünschten Arzneimittelwirkungen in der stationären Altenpflege. MADRIC Endbericht Bd. 18. Bremen

[26] Zaninotto P, Huang YT, Di Gessa G, Abell J, Lassale C, Steptoe A (2020) Polypharmacy is a risk factor for hospital admission due to a fall: evidence from the English Longitudinal Study of Ageing. BMC Public Health 20(1):1804 33243195 10.1186/s12889-020-09920-x PMC7690163

