# Supplementary material for: Medication supply and polypharmacy in long-term care: An overview of possible interventions and the question of a functioning concept
Source: Z Gerontol Geriatr. 2024 Aug 13;58(3):214–9. [Article in German] doi: 10.1007/s00391-024-02340-1 (PMC12048413; doi:10.1007/s00391-024-02340-1)
Supplement: Supplementary file 1 — Dokumentation der Recherche [file 391_2024_2340_MOESM1_ESM.docx]

# Dokumentation Recherche

Suchbegriffe: Polypharmacy, long term care, nursing home, residential care

Einschlusskriterien: Intervention in stationären Pflegeeinrichtungen in D

**Ergebnisse**

**PebMed:** 1.433 Treffer, Filter Clinical Trial und Randomized Controlled Trial: 72 Treffer

Begrenzung auf 20 Jahre (ab 2003-2023): 68 Resultate

Erste Beurteilung anhand der Abstracts, Auswahl der Arbeiten, die sich auf Studien in deutschen Pflegeeinrichtungen beziehen:

| **Artikel** | **Projekttitel/ Akronym** | **Einschluss** | **Ggf. Begründung oder Hinweis** |
| --- | --- | --- | --- |
| Junius-Walker U, Krause O, Thürmann P et al. (2021) Drug Safety for Nursing-Home Residents - Findings of a Pragmatic, Cluster-Randomized, Controlled Intervention Trial in 44 Nursing Homes. Dtsch Arztebl Int 118(42):705–712. doi:10.3238/arztebl.m2021.0297 | HIOPP-3-iTBX | ja | Ergebnisse Intervention |
| Krause O, Wiese B, Doyle I-M et al. (2019) Multidisciplinary intervention to improve medication safety in nursing home residents: protocol of a cluster randomised controlled trial (HIOPP-3-iTBX study). BMC Geriatr 19(1):24. doi:10.1186/s12877-019-1027-0 | HIOPP-3-iTBX | ja | Studienprotokoll, ggf. für Hintergrundinformationen |
| Löffler C, Drewelow E, Paschka SD et al. (2014) Optimizing polypharmacy among elderly hospital patients with chronic diseases--study protocol of the cluster randomized controlled POLITE-RCT trial. Implement Sci.9:151. doi: 10.1186/s13012-014-0151-7 | POLITE-RCT | nein | Setting Krankenhaus |
| Mahlknecht A, Nestler N, Bauer U, et al. (2017) Effect of training and structured medication review on medication appropriateness in nursing home residents and on cooperation between health care professionals: the InTherAKT study protocol. BMC Geriatr 17(1):24. doi:10.1186/s12877-017-0418-3 | InTherAKT | ja | Studienprotokoll, ggf. Für Hintergrundinformationen |
| Mahlknecht A, Krisch L, Nestler N et al. (2019) Impact of training and structured medication review on medication appropriateness and patient-related outcomes in nursing homes: results from the interventional study InTherAKT. BMC Geriatr 19(1):257. doi:10.1186/s12877-019-1263-3 | InTherAKT | ja | Ergebnisse Interventionsstudie |
| Mortsiefer A, Löscher S, Pashutina Y, et al. (2023) Family Conferences to Facilitate Deprescribing in Older Outpatients With Frailty and With Polypharmacy: The COFRAIL Cluster Randomized Trial. JAMA Netw Open. 6(3):e234723. doi: 10.1001/jamanetworkopen.2023.4723 | COFRAIL | nein | Setting ambulante Versorgung |
| Mueller A, Spies CD, Eckardt R et al. (2020) Anticholinergic burden of long-term medication is an independent risk factor for the development of postoperative delirium: A clinical trial. J Clin Anesth. 61:109632. doi: 10.1016/j.jclinane | PERATECS | nein | Setting Krankenhaus |
| Salm C, Sauer J, Binder N et al. (2022) Over- and under-prescribing, and their association with functional disability in older patients at risk of further decline in Germany – a cross-sectional survey conducted as part of a randomised comparative effectiveness trial. BMC Geriatr 22, 564. https://doi.org/10.1186/s12877-022-03242-w | LoChro | nein | Setting Krankenhaus |
| Stolz R, Krause O, Junius-Walker U et al. (2023) The role of qualification and quality management in the prescription of antipsychotics and potentially inappropriate medication (PIM) in nursing home residents in Germany: results of the HIOPP-3-iTBX study. Aging Clin Exp Res 35(10):2227–2235. doi:10.1007/s40520-023-02513-9 | HIOPP-3-iTBX | ja | Weiterführende Informationen zum Projekt |

**CINAHL**: 911 Treffer, Filter Publikationen ab 2003, continental europe: 93 Treffer (Hinweis: kein Filter Interventionsstudie oder ähnliches)

Erste Beurteilung anhand der Abstracts, Auswahl der Arbeiten, die sich auf Studien in deutschen Pflegeeinrichtungen beziehen:

| **Artikel** | **Projekttitel/ Akronym** | **Einschluss** | **Ggf. Begründung oder Hinweis** |
| --- | --- | --- | --- |
| Dörks M, Schmiemann G, Hoffmann F (2016) Pro re nata (as needed) medication in nursing homes: the longer you stay, the more you get?. Eur J Clin Pharmacol 72, 995–1001. https://doi.org/10.1007/s00228-016-2059-4 |  | nein | Keine Intervention |
| Fassmer AM, Hoffmann F (2020) Acute health care services use among nursing home residents in Germany: a comparative analysis of out-of-hours medical care, emergency department visits and acute hospital admissions. Aging Clin Exp Res 32, 1359–1368. https://doi.org/10.1007/s40520-019-01306-3 |  | nein | Kohortenstudie, keine Intervention |
| Hoffmann F, Boeschen D, Dörks M et al. (2015) Renal insufficiency and medication in nursing home residents—a cross-sectional study (IMREN). Dtsch Arztebl Int 112: 92–8. DOI: 10.3238/arztebl.2016.0092 |  | nein | Prospektive Kohortenstudie, keine Intervention |
| Junius-Walker U, Krause O, Thürmann P et al. (2021) Drug Safety for Nursing-Home Residents - Findings of a Pragmatic, Cluster-Randomized, Controlled Intervention Trial in 44 Nursing Homes. Dtsch Arztebl Int 118(42):705–712. doi:10.3238/arztebl.m2021.0297 | HIOPP-3-iTBX | ja | Ergebnisse Intervention |
| Moßhammer D, Haumann H, Mörike K, Joos S (2016) Polypharmacy--an Upward Trend with Unpredictable Effects. Deutsches Ärzteblatt International 113(38):627-633. doi:10.3238/arztebl.2016.0627 |  | nein | Review |
| Pickenhan L, Schiefermeier-Mach N, Rungg C (2020) Elektrolytdysbalancen - Implikationen für den Berufsstand der Pflege. Pflegewissenschaft 22(1):45-60. doi:10.3936/1712 |  | nein | Thematisch nicht passend, keine Intervention |
| Rausch C, Hoffmann F (2020) Prescribing medications of questionable benefit prior to death: a retrospective study on older nursing home residents with and without dementia in Germany. Eur J Clin Pharmacol 76, 877–885. https://doi.org/10.1007/s00228-020-02859-3 |  | nein | Retrospektive Analyse, keine Intervention |
| Stolz R, Krause O, Junius-Walker U et al. (2023) The role of qualification and quality management in the prescription of antipsychotics and potentially inappropriate medication (PIM) in nursing home residents in Germany: results of the HIOPP-3-iTBX study. Aging Clin Exp Res 35(10):2227–2235. doi:10.1007/s40520-023-02513-9 | HIOPP-3-iTBX | ja | Weiterführende Informationen zum Projekt |
| Wolf-Ostermann K, Schmidt A, Gräske J (2016) Arzneimitteltherapiesicherheit in Einrichtungen der stationären Langzeitpflege. Erste Ergebnisse der MADRIC-Studie. Pflegewissenschaft 18(7/8):398–410 | MADRIC | nein | Nur Baseline-Erhebung >> Ergebnisse Recherchieren!! |
| Virnau L, Braesigk A, Deutsch T et al. (2022) General practitioners' willingness to participate in research networks in Germany. Scand J Prim Health Care 40(2):237–245. doi:10.1080/02813432.2022.2074052 |  | nein | Befragung, Keine Intervention |

**Gezielte Suche anhand Hinweise aus Primärrecherche (Google, Google Scholar):**

| **Suchbegriff** | **Auslöser** | **Identifizierte Publikation** | **Einschluss** | **Ggf. Begründung oder Hinweis** |
| --- | --- | --- | --- | --- |
| MADRIC | Daten zu Baslineerhebung | Wolf-Ostermann K, Schmidt A, Gräske J (2016) IT-gestütztes Monitoring von unerwünschten Arzneimittelwirkungen in der stationären Altenpflege. MADRIC Endbericht, Bd 18, Bremen | ja | Ergebnisse Interventionsstudie |
| Arzneimitteltherapiesicherheit, AMTS-Pfleger, AMTS-Ampel | Hinweise Studiendesign HIOPP-3-iTBX (Krause et al. 2019) | Thürmann P, Jaehde U (2016) ArzneiMitteltherapiesicherheit bei Patienten in Einrichtungen der Langzeitpflege (AMTS-AMPEL). Eine prospektive Interventionsstudie, Wuppertal, Bonn | ja | Ergebnisse Interventionsstudie |
|  |  | Thürmann P, Jaehde U (2011) Arzneimitteltherapiesicherheit in Alten- und Pflegeheimen: Querschnittsanalyse und Machbarkeit eines multidisziplinären Ansatzes. Abschlussbericht Bundesministerium für Gesundheit. | ja | Ergebnisse Pilotstudie |

Ergänzende Suche nach Forschungsprojekten mit dem Thema Medikamentenmanagement in der Langzeitpflege, gefördert durch den Gemeinsamen Bundesausschuss (Innovationsfond-Förderung): <https://innovationsfonds.g-ba.de/>

Gefundenes Projekte: Langenberger B, Vogt V, Busse R, siegel M (2023) Optimierte Arzneimittelversorgung für pflegebedürftige geriatrische Patienten. Evaluationsbericht gemäß Nr. 14.1 ANBest-IF

# Eingeschlossene Studien Überblick

| **Artikel** | **Projekttitel/ Akronym** | **Design** | **Intervention** | **Stichprobe** | **Endpunkte & Ergebnisse** |
| --- | --- | --- | --- | --- | --- |
| Thürmann & Jahede 2011 | Arzneimitteltherapie in Alten- und Pflegeheimen | prospektive Querschnittsanalyse, Pilotstudie | Entwicklung auf Basis der Ergebnisse einer prospektiven Querschnittanalyse:   - strukturierte Fortbildungsangebote für Ärzt*innen, Schulungen für Pflegende sowie den heimversorgenden Apotheker*innen zu UAW und AbP - Etablierung eines AMTS Teams, - Entwicklung einer AMTS Karte | - Prospektive Querschnittsanalyse (für Entwicklung der Intervention): 11 Pflegeheime, - N= 778 Bewohner*innen; - Interventionskohorte:   4 Pflegeheime,   - N = 42 Bewohner*innen | - Machbarkeit der Intervention: wird als umsetzbar eingeschätzt, Schwierigkeiten beim Einbinden der Hausärzte*innen - UAW Interventionskohorte: 30-Tages-Prävalenz 15,93, die Inzidenz 7,62 pro 100 Heimbewohnermonate |
| Thürmann & Jahede 2016 | ArzneiMitteltherapie-sicherheit bei Patienten in Einrichtungen der Langzeitpflege (AMTS-AMPEL) | Einarmige Interventionsstudie (Vorher-Nachher Vergleich) | Optimierte Intervention aus Vorgängerprojekt:   - Fortbildungen Hausärzte*innen, Pflegekräfte, heimversorgende Apotheken - AMTS-Teams - AMTS-Merkkarte | - 8 Pflegeheime, - drei Messzeitpunkte - N_0_ = 334; - N_6 Monate_ = 296; - N_12 Monate_ = 273 | - Vermeidbare und nicht vermeidbare UAW: weniger vermeidbare UAW nach Intervention, Anstieg unvermeidbarer UAW |
| Junius-Walker et al. 2021 | HIOPP-3-iTBX-Studie | Cluster-randomisierte Interventionsstudie | - Medikationsreviews durch spezifisch geschulte Apotheker*innen, - Fortbildungen für Pflegekräfte und Hausärzt*innen, - eine Toolbox zur AMTS (Entlass- sowie Visitentool, AMTS-Karte etc.) und Changemanagementseminare für Pflegende, Ärzt*innen und Apotheker*innen | - 44 Pflegeheime (23 IG/ 21 KG) - IG N = 452 Bewohner*innen, - KG N = 410 Bewohner*innen | - Primär: Anzahl PIM und/oder 2 Neuroleptika; - Sekundär: Sturzinzidenz, Hospitalisierungen, Lebensqualität, Gesundheitskosten - Ergebnis: keine signifikanten Unterschiede zw. IG und KG |
| Wolf-Ostermann et al. 2016 | MADRIC | Längsschnittstudie mit IG und KG (keine Randomisierung) | - IT-gestütztes Monitoring System zur Vermeidung von UAW | - 2 Pflegeheime (1 IG, 1 KG), - IG N = 48 - IG K = 72 | - Gesundheitsbezogene Lebensqualität, Pflegebedarf, Aktivitäten des täglichen Lebens, Kognitive Fähigkeiten - Keine signifikanten Unterschiede der beiden Gruppen |
| Mahlknecht et al. 2019 | InTherAKT | Einarmige Interventionsstudie (Vorher-Nachher Vergleich) | - Systematische Weiterbildung aller beteiligter Berufsgruppen - Einführung eines strukturierten Medikations-Reviews (multiprofessionell) | - 9 Pflegeheime - N = 83 Bewohner*innen | - Primär: Einschätzung Angemessenheit der Medikation (Medication Appropriateness Index = MAI) - Sekundär: Kognition, Delir, Agitation, Mobilität, Anzahl Medikamente, schwerwiegende Arzneimittel-Interaktionen, angemessene Analgetika Verschreibung - Keine signifikanten Veränderungen nach Intervention, nur weniger Agitation - Gruppe mit initial hohen MAI mit verbessertem Score |
| Fahrentholz et al. 2023 | Optimierte Arzneimittelversorgung für pflegebedürftige geriatrische Patienten (OAV) | Zweiarmige, sektorenübergreifende, nicht randomisierte Interventionsstudie (retrospektive Bildung KG mit Krankenkassen-daten) | - Kooperation von Pflegekräfte, Ärzte*innen, Apotheker*innen - EDV gestütztes Risikomanagement zur Verbesserung der Medikamentenversorgung ambulant und stationär versorgter pflegebedürftiger Personen | - IG N = 1.325 (110 ambulant, 1215 stationär versorgt) - KG N (gematcht anhand IG) = 2.438 (225 ambulant, 2.258 stationär versorgt) | - Primär: UAW - Sekundär: Kranken-hauseinweisungen, Anzahl PIM, Polypharmazie - Inzidenz UAW und Krankenhaus-einweisungen signifikant gesenkt - Bei Bewohner*innen der Langzeitpflege außerdem Senkung verordneter PIM und Anteil mit Polypharmazie |
|  | | | | | |
| AbP = Arzneimittel-bedingte Probleme; IG = Interventionsgruppe; KG = Kontrollgruppe; MAI = Medication Appropriateness Index; PIM = potentiell inadäquate Medikamente; UAW = Unerwünschte Arzneimittelwirkung; AMTS = Arzneimitteltherapiesicherheit | | | | | |
